# Fully Immersive Virtual Reality Game-Based Training for an Adolescent with Spastic Diplegic Cerebral Palsy: A Case Report

**DOI:** 10.3390/children9101512

**Published:** 2022-10-03

**Authors:** Kyeongbong Lee, HyeJin Oh, GyuChang Lee

**Affiliations:** 1 Department of Physical Therapy, Kangwon National University, Samcheok 25949, Korea; 2 Department of Physical Therapy, Graduate School, Kyungnam University, Changwon 51767, Korea; 3 Department of Physical Therapy, Kyungnam University, Changwon 51767, Korea

**Keywords:** cerebral palsy, fully immersive virtual reality, case report

## Abstract

Background: Recently, virtual reality-based training (VR-based training) is receiving attention as greater emphasis is placed on the importance of interest and motivation in participation. However, studies investigating the effects of fully immersive VR-based training are insufficient. Case presentation: We report a case of using a fully immersive VR game-based training in a patient with cerebral palsy. A 15-year-old girl was diagnosed with spastic diplegia cerebral palsy Gross Motor Function Classification System level II. A six-week intervention (18 sessions) phase was performed with one fully immersive VR game using PlayStation^®^VR in three sessions per week. After 18 sessions of training, the scores on the gross motor function measure-88 (Gross Motor Function Measure-88-GMFM-88), pediatric balance scale (PBS), timed up and go test (TUG), functional gait assessment (FGA), and 10 m walking test (10MWT) were improved: GMFM-88, 91.56 points (9.31 points increase); PBS, 45 points (6 points increase); TUG, 8.23 s (6.9 s decrease); FGA, 11 points (3 points increase); the 10 MWT, 5.27 s (6.59 s decrease). Conclusions: This study found that a fully immersive VR game-based training using PlayStation^®^VR may be an effective intervention for GMFCS level II adolescent, leading to some improvement of motor function, balance and gait skills in adolescents with cerebral palsy.

## 1. Introduction

Cerebral palsy (CP) is a nonprogressive motor impairment syndrome caused by brain defects or lesions that occur in an immature brain before or during birth or within two years of birth [1]. It is accompanied by motor disorder; disturbances of sensation, cognition, perception, communication, and/or behavior; and/or seizure disorder and affects overall development [2,3]. Accordingly, children with CP show reduced postural control when engaging in several activities such as sitting, standing, and walking and have limitations in performing the activities of daily living [4,5].

Therapeutic approaches for CP include neurodevelopmental treatment, Vojta therapy, sensory integration therapy, and conductive education. Specific therapeutic strategies for CP may differ, but they all aim to improve the independence of children with CP [6]. Other rehabilitation methods include neuromuscular electrical stimulation [7] and dynamic orthosis [8]. Considering that the rehabilitation period of patients with CP is observed in a prolonged period of time, it is important to induce the sustained participation of these patients, making sure that the therapeutic interventions are interesting and easily performed [9]. Some approaches have limitations in inducing patients’ interests [10,11]. Additionally, since these approaches have insufficient feedback and accurate analysis on the patient’s movements, continuous implementation with incorrect movements can lead to reduced intervention effectiveness due to accumulated errors [12,13]. It is difficult to assess the long-term effects of rehabilitation because ways on how to assess whether the interventions performed at the hospitals are correctly performed at home are not yet available [12].

Recently, virtual reality-based training (VR-based training) is receiving attention as greater emphasis is placed on the importance of interest and motivation in participation. VR refers to interactive simulation that allows a user to experience reality through computer hardware and software [14]. VR creates an environment that provides a user experience similar to the real world through a computer to enhance the user’s interest and motivation for training [15]. Moreover, VR increases the users’ own motivation [13]. In VR, a user shows realistic responses such as moving and manipulating objects and performing fixed tasks [16]. When a specific task is expressed in a game form, the feedback of VR responds to visual and audio information of the task, informs of the user’s errors in movement, and effectively controls the movement [11,13]. Thus, VR-based training is an intervention approach that is considered superior compared to the traditional interventions, which are passive and repetitive and do not provide sensory feedback on the outcome of performance [17].

Clinicians rate motivation as the most influential personal characteristic that determines motor and functional outcomes in children with CP [18,19]. As a result of examining factors affecting the acquisition of motor skills in children with CP targeting physical therapists, one of the important factors influencing the acquisition of motor skills in children with CP was motivation [18]. Also, in a study on family-centered functional therapy for children with CP, motivation was an important factor influencing motor and functional outcomes of children with CP [19]. Evidence from the neuroplasticity literature has confirmed that motivation is an important modulator of functional plasticity. Neural reorganization occurs at the molecular and behavioral levels and responds to factors such as development, environment, disease, and therapy [20]. Theoretically, therapists can use motivation to shape sensorimotor environments to enhance reorganization and optimize rehabilitation outcomes through enhanced engagement [21]. Particularly in adolescence, developmental changes in primary motivation circuitry may promote novelty-seeking behavior and augment incentive motivational processes [22].

Technological changes in the field of video games over the past few years make it possible for people to play several video games in a three-dimensional (3D) VR environment instead of a two-dimensional VR environment, or even in a VR environment using a head-mounted display (HMD) [23]. Most VR systems lack the ability to capture the user’s full range of motion, limiting their ability to fully immerse the user in the virtual environment. In contrast, fully immersive virtual reality allows for full mobility by capturing human motion and reproducing the same motion in the virtual environment [24,25].

As such, the effects of VR-based training have been verified in previous studies, but most of the studies have investigated the effects of semi-immersive VR-based training. Different from fully immersive VR, semi-immersive VR is a method of performing movement and playing games while watching a television or computer monitor screen [26]. Semi-immersive VR offers less immersion and interaction than fully immersive VR [26]. However, studies investigating the effects of fully immersive VR-based training are insufficient. There is specifically no study conducted in adolescents with CP. Therefore, this study aimed to investigate the effect of fully immersive VR game-based training on motor function, balance, and gait ability when it is applied to an adolescent with CP and to confirm the beneficial effects of fully immersive VR game-based training as a therapeutic approach for CP. The hypothesis of this study is that fully immersive VR game-based training will have a positive effect on motor function, balance and gait ability when applied to adolescents with CP.

## 2. Description of the Case

### 2.1. History and Systematic Review

The subject was a 15-year-old female adolescent (height, 160 cm; weight, 45 kg) who had been diagnosed with spastic diplegic CP and has been receiving treatment at H Rehabilitation Hospital in the Republic of Korea as an outpatient. As the second twin, the subject was delivered through a cesarean section at the gestational age of 32 weeks and 5 days. The subject’s mother received inpatient treatment from 32 weeks of gestation due to gestosis and gave birth during her hospital stay. Since the subject’s birth weight was 1.82 kg, she was placed in an incubator for 2 days before discharge. She developed without any major problems but started to show difficulties in standing and walking alone at 10 months, excessively relying on the support of her upper limbs when standing and walking. The subject underwent magnetic resonance imaging (MRI) and electroencephalography (EEG), but significant findings were not observed based on MRI and EEG results. However, the subject showed neck and proximal instability and difficulties in postural control and movement of her pelvic and lower extremity. She was diagnosed with spastic diplegic CP at 12 months. Her main problems were developmental delays and difficulty controlling leg movements. Her major problem was the difficulty in controlling her trunk and leg movements due to low muscle tone in her trunk. To solve these problems, she was referred to outpatient physical therapy and has been receiving physical therapy twice a week since 12 months. The patient and their family’s goal for physical therapy is to independently climb up and down stairs by improving the balance ability during standing and walking.

The examination of the subject before the intervention are as follows (Table 1).

The muscle tone of the subject with spastic diplegia was very mild, scoring 0 to 1+ by the modified Ashworth Scale under the same testing positions as in the study by Bohannon and Smith [27]. The subject had deficient postural control due to trunk impairment. Based on the Gross Motor Function Classification System (GMFCS), the subject was categorized with GMFCS level II since she could walk without support in most settings. However, she needed assistance in certain situations (such as uneven terrain, inclines, and long distances). She used ankle-foot orthoses (AFOs) for walking long distances. She had difficulty with running and jumping. She was able to climb up and down stairs holding a railing or with physical assistance if there was no railing. She had limitations in the performance of gross motor skills and needed adaptations to enable participation in physical activities and sports. The subject showed forward head posture in sitting and standing positions; was unable to position her neck at midline; had thoracic kyphosis, scapular protraction, and rounded shoulders; and experienced shoulder joint internal rotation. The subject used the left hand dominantly. The subject had difficulty in weight bearing and weight transfer left or right in a standing position, and weight bearing to the left was dominant compared to the right. Due to miserable malalignment syndrome, femoral excessive anteversion, genu valgum, tibial lateral torsion, subtalar joint hyper pronation, and pronated foot were observed in this subject. Contracture and deformity in the lower extremity were observed as the subject was unable to maintain correct posture and movement. There was a limitation to the range of joint motion in knee extension, ankle dorsiflexion, and foot inversion. The subject had no difficulty in performing tasks using her hands. She possessed the necessary speed and accuracy on the manual ability assessment without any limitation in performing the activities of daily living. She was independent with activities of daily living (ADLs). She could communicate sufficiently regardless of who the communication partner was. The subject had no impairments of visual, hearing, somatosensory, proprioception, kinesthesia, or vestibular function. The subject had neither psychological problems nor any problems in the cardiopulmonary, gastrointestinal, endocrine, urinary, and reproductive systems. The subject had no history of seizures, surgery, or botulinum toxin injections within the past 3 years.

### 2.2. Tests and Measures

In this study, the Gross Motor Function Measure-88 (GMFM-88) was used to measure gross motor function changes. The Pediatric Balance Scale (PBS) and the Timed Up and Go (TUG) test were used to test balance, and the Functional Gait Assessment (FGA) and 10-Meter Walking Test (10MWT) were used to assess gait. The subject’s gross motor function, balance, and gait were assessed 19 times in total, before intervention and after 18 sessions of intervention.

#### 2.2.1. Gross Motor Function Measure-88 (GMFM-88)

The GMFM-88 is the most commonly used tool for examining gross motor function in CP and Down syndrome [28,29]. This tool showed high validity at 0.91 when applied to children with CP [30]. Additionally, the GMFM-88 was reported to be a useful method for measuring gross motor function in children with CP because of its high reliability, with inter-rater reliability of 0.77, test–retest reliability of 0.88, and intra-rater reliability of 0.68 [31].

#### 2.2.2. Pediatric Balance Scale (PBS)

The PBS is a standardized tool for testing balance. Franjoine et al. [32] developed the tool by modifying the Berg Balance Scale to test the functional balance of the school-age population with mild-to-moderate motor impairment. The tool has been confirmed to be reliable in terms of both intra-rater reliability (intraclass correlation coefficient (ICC) = 0.99) and inter-rater reliability (ICC = 0.99). The items can be measured within 15 min and do not require the use of specialized equipment [32].

#### 2.2.3. Timed up and Go Test (TUG)

The TUG test is a tool that can be used to quickly assess the functional mobility and balance. The test showed high intra-rater reliability (r = 0.99) and high inter-rater reliability (r = 0.98) [33].

#### 2.2.4. Functional Gait Assessment (FGA)

The FGA is a dynamic gait index comprising 10 items. The FGA shows high reliability and validity for stroke patients and is found to have high correlation with other gait and balance assessment tools (Rivermead Mobility Index, Berg Balance Scale, Functional Ambulatory Category) with Spearman rho values between 0.71 and 0.93 [34].

#### 2.2.5. 10-Meter Walking Test (10MWT)

The 10MWT is a test used to assess walking speed by measuring the time taken to walk a 10 m distance at a safe speed. The test–retest reliability of the 10MWT has been reported to be excellent (ICC = 0.93) [35].

### 2.3. Clinical Impression

Before intervention, the subject of this study was able to walk independently but had difficulty in performing active functions such as standing on one foot, walking up and down steps, running, and jumping. Therefore, the subject’s test and measure results revealed the following: Gross Motor Function Classification System level, Level 2; GMFM-88 score, 82.25 points; PBS score, 39 points; TUG test score, 15.13 s; FGA score, 8 points; and 10MWT score, 11.88 s.

### 2.4. Intervention

In this study, training was applied to the subject using PlayStation^®^VR, a fully immersive VR system. PlayStation^®^VR is a headset that is connected to the PlayStation 4 for use. When a user wears the headset, a 360-degree omnidirectional 3-dimensional (3D) space is created surrounding the user. The HMD works as movements are sensed through the two motion-sensing hand controllers and the motion-sensing camera in front of the headset. Using the headphones (with earpieces) connected to the headset, the user can experience enhanced realistic sensation by connecting the headphones to the 3D audio technology.

**Figure 1:**
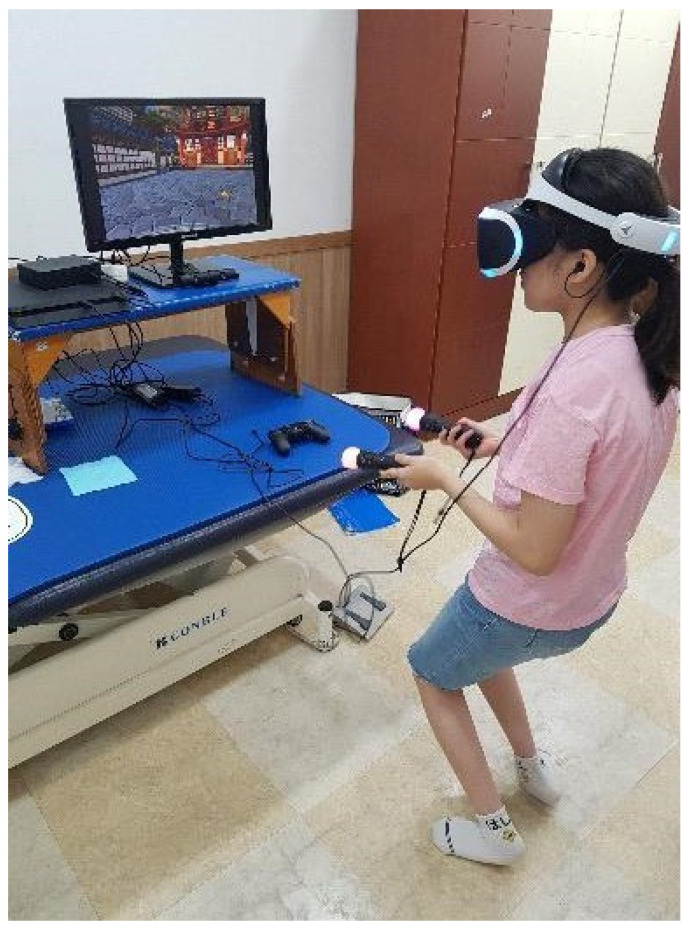
Fully immersive VR game-based training.

The PlayStation^®^VR (PlayStation^®^VR, Sony, Tokyo, Japan) for fully immersive VR game-based training was installed in an indoor space free of external disturbances. The game played through PlayStation^®^VR was one of the VR-based games with a fixed background called Fruit Ninja (Fruit Ninja, Sony, Tokyo, Japan). Fruit Ninja is a game that involves the player to slice the fruit thrown into the VR scene with a blade controlled by the player’s arms or hands. When a fruit is thrown into the VR scene with a fixed background, the player attempts to slice the fruit in half by performing slicing motions swinging the arms or hands. The player can cut the fruit into several pieces with a single swing, at which time an additional point is awarded. Players must slice all the fruits, and the game is over if they miss three fruits. In this study, the subject maintained a standing posture during the fully immersive VR game-based training. Considering that the subject may experience fatigue, the training was conducted in 3 sets of 10 min games for 30 min in total, with a 5 min rest time between the sets. After the intervention, the subject was provided with a 10 min rest time and was subsequently examined for gross motor function, balance, and gait (Figure 1).

## 3. Results

**Figure 2:**
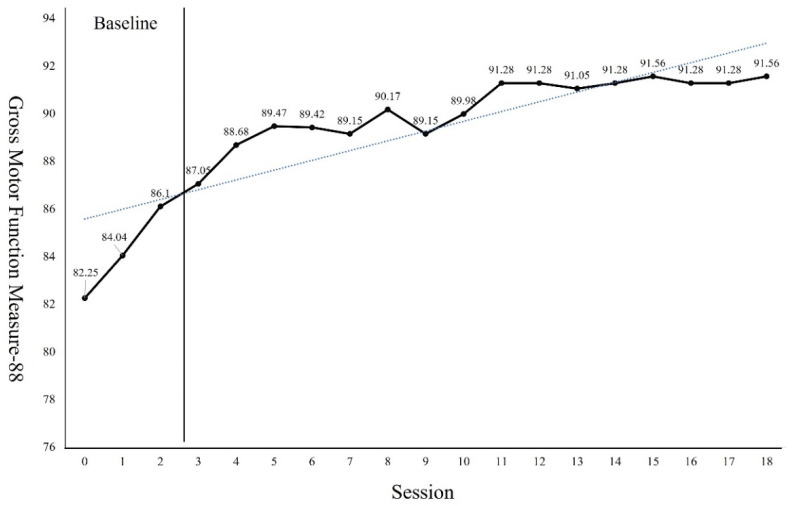
Changes of Gross Motor Function Measure.

**Figure 3:**
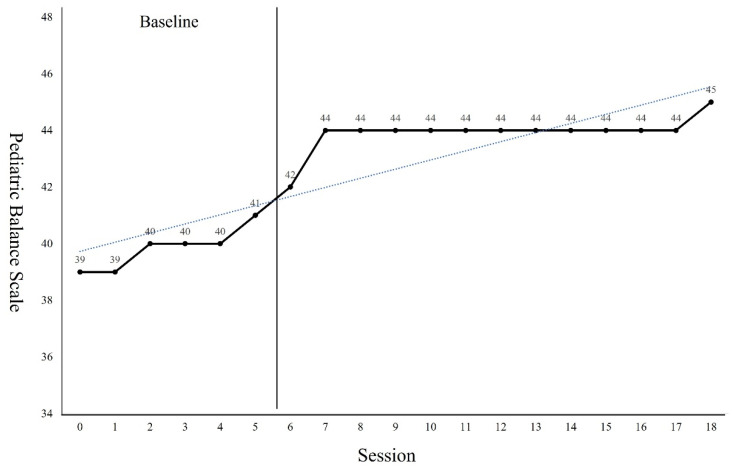
Changes of Pediatric Balance Scale.

**Figure 4:**
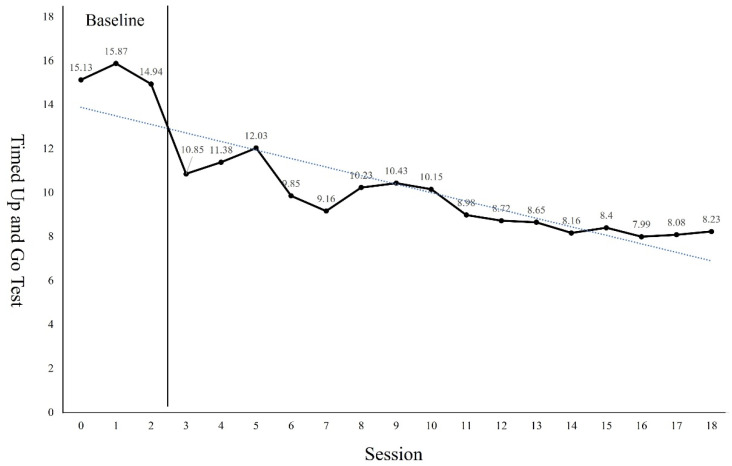
Changes of Timed Up and Go Test.

**Figure 5:**
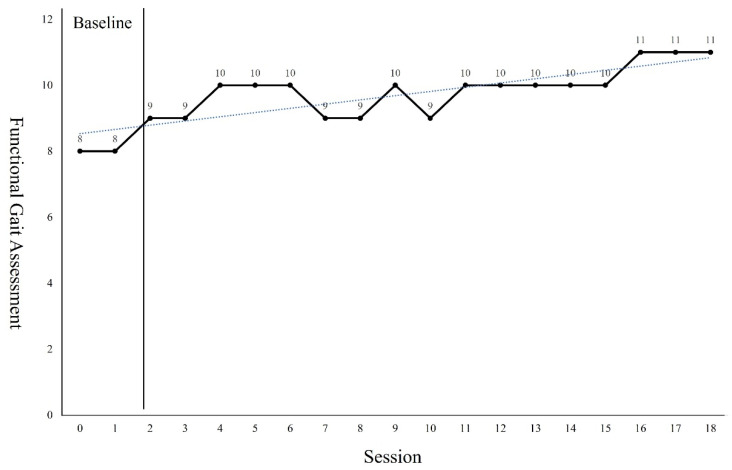
Changes of Functional Gait Assessment.

**Figure 6:**
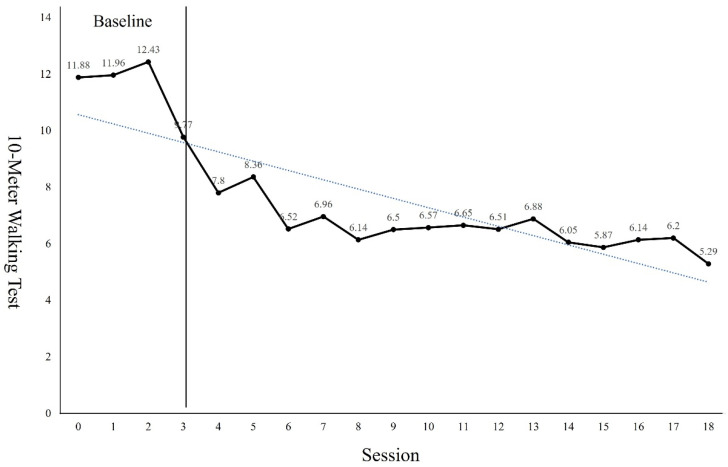
Changes of 10MWT.

The GMFM-88 results showed a 9.31 points increase from the pre-intervention result of 82.25 points after the 18 sessions of intervention (Figure 2). The PBS results showed a 6 points increase from the pre-intervention result of 39 points after the 18 sessions of intervention (Figure 3). Regarding the TUG test, the results showed a 6.9 s reduction from 15.13 s before the intervention to 8.23 s after the 18 sessions of intervention (Figure 4). The FGA results showed a 3 points increase from 8 points before the intervention to 11 points after the 18 sessions of intervention (Figure 5). The 10MWT results showed a decrease of 6.59 s from 11.88 s before the intervention to 5.29 s after the 18 sessions of intervention (Figure 6).

After fully immersive VR game-based training intervention, she was able to better maintain her balance and stability when walking compared to before the intervention. There was no adverse event such as a fall or fractures during the intervention. She was very interested in fully immersive VR game-based training intervention and actively participated in the intervention. She was able to be compliant with all interventions from before pre-intervention until after the 18 sessions of intervention.

## 4. Discussion

In this study, the effect of fully immersive VR game-based training on gross motor function, balance, and gait was investigated when the training was applied to an adolescent diagnosed with spastic diplegic CP. As a result, improvements were observed based on the results of the GMFM-88, PBS, TUG test, 10MWT, and FGA with the implementation of fully immersive VR game-based training. These results suggest that fully immersive VR game-based training can have positive effects on gross motor function, balance, and gait in adolescents with spastic diplegic CP.

VR is an interactive, immersive and simulated experience in an environment and real-world objects created by computer systems responding to real-time user movements [36,37]. VR can be offered in an immersive and non-immersive manner. To achieve a more natural experience, immersive VR simulations are often generated using an HMD [38,39,40] or room-scale displays that surround the user such as the CAVE [41,42]. In both cases, immersion and presence are heightened when the motion of the visual field is properly linked with the motion of the head [43]. In this study, a fully immersive VR game-based training was applied as an intervention using PlayStation^®^VR. PlayStation^®^VR (PlayStation^®^VR, Sony, Tokyo, Japan) is a VR HMD released by Sony Interactive Entertainment for PlayStation 4.

VR-based interventions have been used in the treatment of patients with CP [44,45]. Rehabilitation through VR enables multiple levels of complex training with feedback in a safe and controlled environment [46,47]. VR also allows clinicians to control the duration and intensity of tasks not performed in the real world [48]. At the same time, VR provides encouragement and motivation during rehabilitation, providing a meaningful learning experience [46]. However, one of the major drawbacks of VR is cybersickness [49,50,51]. Cybersickness is characterized by bodily discomfort associated with exposure to VR content [50]. Factors that cause cybersickness in VR can be classified into the following three categories: contents, hardware, and user’s personal characteristics [52]. In this study, it was difficult to determine the occurrence and extent of cybersickness considering the factors that cause cybersickness in terms of hardware because studies on HMD-based fully immersive VR intervention have rarely been reported. Regarding a user’s personal characteristics, the possibility of cybersickness could not be excluded in this study since the intended subject, an adolescent with CP, belongs to a vulnerable group of young people with disabilities. Thus, considering the possibility of such side effects, this study was conducted as a case report of one person instead of many.

Arnoni et al. [53] reported that, as a result of applying VR game-based training to children with CP, GMFCS I-II, gross motor function was improved. Ökmen et al. [54] reported an improvement in motor functions in the study group in which VR therapy was additionally applied to conventional treatment as a result of the comparative study of the effects of VR therapy through a randomized controlled study in children with CP. The additional application of VR therapy to conventional treatment may have a significant effect on improving motor functions by using various sports games in a sitting position. Ghai, S. and Ghai, I. [55] suggested through meta-analysis that VR training had a positive effect on improving gait performance in CP. Spatiotemporal parameters such as gait velocity, cadence, and stride length improved after VR training for at least 20–30 min each time, 4 times a week or more, and for a total of 8 weeks or more. Pourazar et al. [56] reported improvement in dynamic balance as a result of applying VR game-based training to children with spastic hemiplegic cerebral palsy. Also, posture control training in the sitting position using a VR training program was found to be more effective in improving the sitting balance and trunk stability of children with CP [57]. As such, various intervention methods using VR games and positive effects have been suggested; it is thought that VR games aimed at improving trunk and postural stability induced gross motor and gait parameters. The fully immersive VR game-based training used in the present study required various movements in a standing position; it seems to have had a positive effect on improving the stability of the trunk. As a result, it is considered that the subject of this study also enhanced postural stability, essential for gait ability, and eventually the subject’s gait performance was improved.

As can be seen from the trend line in Figures, the first 4 to 5 sessions showed significant improvement in the GMFM-88, TUG, and 10MWT. However, in PBS, a significant improvement was observed in 4 to 7 sessions. In the case of the GMFM-88, TUG, and 10MWT, it is thought that immediate changes were possible from the early session because quantitative aspects were examined rather than qualitative aspects. On the other hand, PBS, which examines the balance in more detail, seems to have changed from the 4th session as the quality improved after a certain amount of training was repeated. In addition, the FGA consists of items that require more postural control, balance ability, and gross motor function than her examination tools. Because the number of items to be examined was small, it seems that it was difficult to see a large change within a short session.

In the present study, the outcome measures were selected to find the minimal important differences in the subject’s balance and gait. Spastic diplegic CP individuals are usually more affected in function of lower extremity than by upper extremity. Since the subject in the present study was a spastic diplegic CP adolescent, we tried to focus on improving motor function, balance and gait over a function of upper extremity. A training fully immersive VR game was applied as an intervention in a standing position to improve balance and gait ability with the subject’s concentration improved. The subject’s concentration on rehabilitation intervention was improved on this point, and it is considered that the subject’s balance and gait ability were enhanced by inducing improvement of postural stability and standing balance due to performing various posture and movement controls in the standing position.

The advantages of VR in rehabilitation are related to the simultaneous stimulation of cognitive and motor processes [58,59]. VR enables a rewarding and challenging virtual environment to engage in new experiences in both recreational and fun contexts [58,60]. In addition, VR encourages and motivates them to solve problems in a variety of situations. It also promotes neuroplasticity by providing social engagement, flexibility of use, feedback, and repetitive task performance of varying degrees of difficulty [61,62,63]. Therefore, in this study, it is thought that the improvement in motor function, balance, and gait of the subject after a fully immersive VR game-based training was obtained by the above mechanism.

However, this study has some limitations. First, this study lacks quality because it is a case report of a single subject. Naturally, the effects of fully immersive VR game-based training cannot be generalized based on the findings of this study alone. Second, in addition, in this study, the subject participated in the same intervention three times a week for six weeks, a total of 18 times, and participated in outcome measures including motor function, balance, and gait ability a total of 19 times. In particular, participation in the same tests and measures several times may threaten the reliability of the outcome measures due to the practice effect or memory effect. Third, this study did not use a variety of game content. In fact, this study initially investigated two games: a VR game with a fixed background called Fruit Ninja and a VR game with a moving background called Driveclub. As a result of applying the two games in a preliminary study, the VR game with a moving background led to increased sway velocity and sway length and more cybersickness than the VR game with a fixed background. However, the VR game with a fixed background caused no sway of balance in standing position and relatively less cybersickness than the VR game with a moving background and, thus, indicated fewer side effects. Therefore, to reduce the risk of accompanying cybersickness, this study used the VR game with a fixed background and thus failed to cover a wider variety of game contents. Fourth, in this study, more different types of VR equipment were not applied. Lastly, the long-term effects of the fully immersive VR game-based training were not investigated. Therefore, future studies should apply various game contents with progressive intensity to more subjects to verify the effect of fully immersive VR game-based training on adolescents with CP, and it should apply more various types of VR equipment and compare its effects. A high-quality clinical study, such as randomized controlled trials, will be needed to compare the therapeutic effects. In addition, it is necessary to investigate whether the effects of fully immersive VR game-based training persist in the long term through a long-term study.

## 5. Conclusions

In this study, the effects of fully immersive virtual reality game-based training on gross motor function, balance, and gait were investigated when it is applied to an adolescent with cerebral palsy. As a result, it was found that fully immersive virtual reality game-based training can have positive effects on gross motor function, balance, and gait in adolescents with cerebral palsy. However, this study is limited in that it involved only one subject, did not apply more diverse games to reduce the risk of cybersickness, and did not apply and compare different types of virtual reality equipment. Therefore, it is necessary for future studies to remedy these issues and verify the effects of fully immersive virtual reality game-based training on adolescents with cerebral palsy by conducting higher-quality studies.

## Figures and Tables

**Table 1:** Basic physical function of the subject.

Examination	Grade or Descriptions
Muscle tone by MAS	0 to +1
Motor function by GMFCS	Level II
LOM	Knee extension, Ankle dorsiflexion and inversion
Postures	Forward head posture in sitting and standing positions was unable to position her neck at midline had thoracic kyphosis, scapular protraction, and rounded shoulders; and experienced shoulder joint internal rotation. had difficulty in weight bearing and weight transfer left or right in a standing position Due to miserable malalignment syndrome, femoral excessive anteversion, genu valgum, tibial lateral torsion, subtalar joint hyperpronation, and pronated foot

Abbreviations: MAS, Modified Ashworth Scale; GMFCS, Gross Motor Function Classification System; LOM, Limited range of motion.

## Data Availability

The datasets used and/or analyzed during the current study are available from the corresponding author on reasonable request.

## Abbreviations

Cerebral palsy	CP
Virtual Reality	VR
virtual reality-based training	VR-based training
Gross Motor Function Measure-88	GMFM-88
Pediatric Balance Scale	PBS
Timed Up and Go	TUG
Functional Gait Assessment	FGA
10-Meter Walking Test	10MWT
